# Economic Evaluation of a Pre-Hospital Protocol for Patients with Suspected Acute Stroke

**DOI:** 10.3389/fpubh.2018.00043

**Published:** 2018-03-05

**Authors:** Suman Lahiry, Christopher Levi, Joosup Kim, Dominique A. Cadilhac, Andrew Searles

**Affiliations:** ^1^Centre for Clinical Epidemiology and Biostatistics (CCEB), Community Medicine and Clinical Epidemiology, School of Medicine and Public Health (SMPH), Hunter Medical Research Institute (HMRI), University of Newcastle, Callaghan, NSW, Australia; ^2^Sydney Partnership for Health Education Research and Enterprise (SPHERE), Medicine, University of New South Wales, Sydney, NSW, Australia; ^3^Translational Public Health and Evaluation Division, Stroke and Ageing Research, School of Clinical Sciences, Monash University, Melbourne, VIC, Australia; ^4^Public Health, Stroke Division, the Florey Institute of Neuroscience and Mental Health, University of Melbourne, Heidelberg, VIC, Australia; ^5^Faculty of Health and Medicine, University of Newcastle, Callaghan, NSW, Australia; ^6^Health Research Economics, Hunter Medical Research Institute (HMRI), New Lambton, NSW, Australia

**Keywords:** stroke, acute stroke, economic evaluation, pre-hospital protocol, thrombolysis

## Abstract

**Background:**

In regional and rural Australia, patients experiencing ischemic stroke do not have equitable access to an intravenous recombinant tissue plasminogen activator (tPA). Although thrombolysis with tPA is a clinically proven and cost-effective treatment for eligible stroke patients, there are few economic evaluations on pre-hospital triage interventions to improve access to tPA.

**Aim:**

To describe the potential cost-effectiveness of the pre-hospital acute stroke triage (PAST) protocol implemented to provide priority transfer of appropriate patients from smaller hospitals to a primary stroke center (PSC) in regional New South Wales, Australia.

**Materials and methods:**

The PAST protocol was evaluated using a prospective and historical control design. Using aggregated administrative data, a decision analytic model was used to simulate costs and patient outcomes. During the implementation of the PAST protocol (intervention), patient data were collected prospectively at the PSC. Control patients included two groups (i) patients arriving at the PSC in the 12 months before the implementation of the PAST protocol and, (ii) patients from the geographical catchment area of the smaller regional hospitals that were previously not bypassed during the control period. Control data were collected retrospectively. The primary outcome of the economic evaluation was the additional cost per disability adjusted life years (DALYs) averted in the intervention period compared to the control period.

**Results:**

The intervention was associated with a 17 times greater odds of eligible patients receiving tPA (adjusted odds ratio, 95% CI 9.42–31.2, *p* < 0.05) and the majority of the associated costs were incurred during acute care and rehabilitation. Overall, the intervention was associated with an estimated net avoidance of 93.3 DALYs. The estimated average cost per DALY averted per patient in the intervention group compared to the control group was $10,921.

**Conclusion:**

Based on our simulation modeling, the pre-hospital triage intervention was a potentially cost-effective strategy for improving access to tPA therapy for patients with ischemic stroke in regional Australia.

## Introduction

Reperfusion with thrombolysis [intravenous recombinant tissue plasminogen activator, (tPA)] within the approved treatment window of 4.5 h from symptom onset is an important component of organized acute ischemic stroke treatment (1, 2). tPA is proven to be a cost-effective therapy when given according to guidelines (3–6), however, the implementation of tPA varies substantially both within and across countries (Table A1 in Appendix).

In Australia, only 3% of patients with ischemic stroke received thrombolysis according to the 2009 national audit, improving to 7% by 2014 (7). However, the reported national averages mask the extent of geographical variation in implementation. The few reports available from regional hospitals suggest that access to tPA for stroke in the smaller regional and rural locations can be minimal to non-existent (8, 9). In such situations, new models of care are needed to improve both pre-hospital assessment for potential tPA eligibility and the time between stroke event and treatment in a specialized stroke center.

**Figure A1:**
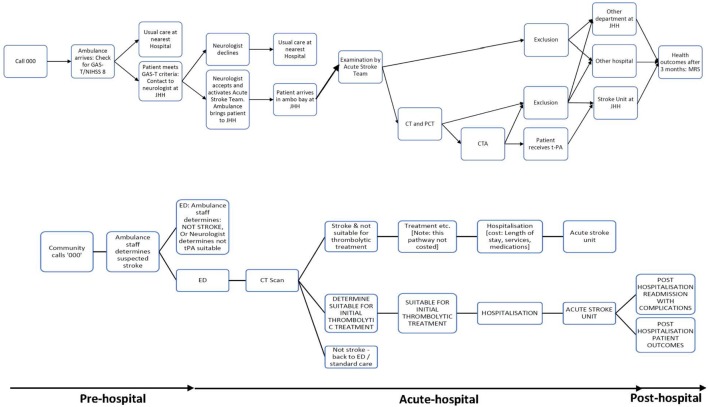
Patient flowchart for acute stroke care pathway using pre-hospital acute stroke triage (PAST) protocol compared to pre-PAST traditional management.

In the Hunter region of New South Wales, Australia, the Pre-Hospital Acute Stroke triage (PAST) protocol was designed to improve access to tPA for eligible patients (Figure A1). The PAST protocol is comprised of: (i) a pre-hospital stroke assessment tool, (ii) an ambulance hospital-bypass protocol for thrombolysis-eligible patients outside the primary catchment area of the regional primary Stroke Centre, John Hunter Hospital (JHH), and (iii) pre-notification and rapid deployment of the JHH’s multidisciplinary stroke team (8, 10).

The PAST protocol was implemented in September 2006 (8, 10) and resulted in a significant increase in the proportion of patients accessing tPA in the JHH and a significant reduction in median times from symptom onset to stroke unit admission (10). Although the PAST protocol and a number of other similar pre-hospital acute stroke triage interventions have demonstrated enhanced tPA implementation (10, 11), these models of care have rarely undergone economic evaluation. Economic evaluations of such programs assist informed decisions about the allocation of available resources and thus facilitate efficient priority setting. Cost-effective models of care that improve access to tPA for eligible patients also have the ability to improve health equity by ensuring those disadvantaged, because of distance from regional stroke centers, have improved access to effective therapy.

### Aims

The aims of this study were to assess overall changes in associated healthcare resource utilization as a consequence of implementing the PAST protocol and to describe the potential cost-effectiveness of the PAST protocol.

## Materials and Methods

### Study Design

The economic analysis was based on observational data with a historical control design. A decision analytic model with simulation was used to calculate the costs and outcomes (initially developed by author DAC). Routinely collected data were analyzed for different periods. The intervention period for the evaluation was commenced on September 14th, 2006.

The principal control cohort was defined as patients presenting to the JHH between September 14th, 2005 and August 31st, 2006. Since, we also needed to capture information for patients who would previously have not been transferred to JHH, data from a second control cohort of patients equivalent to those who were bypassed to JHH in the intervention period (referred to as the alternate pathway) were also obtained. The patients in the alternate pathway (our counterfactual scenario) were assumed not to have received tPA or care in a stroke unit because they would not have been directed to the JHH, with its Stroke Centre, in the control period. Without a specialized Stroke Centre at their local hospital, patients attending these hospitals would not have had access to tPA. The alternative pathway group is independent of the intervention and historical control cohorts. It is a simulated situation for this real-world study using aggregated and de-identified administrative data from the same time period for patients who were managed in hospitals other than JHH with stroke.

Patients included in the intervention period were those presenting to the JHH between September 14th, 2006 and October 30th, 2009, when the PAST protocol was operational and under audit and evaluation. Detailed data were prospectively collected during the intervention period. Where required, the evaluation data were supplemented with local administrative information and imputed using the decision analytic model developed for this study.

### Ethics

The Hunter New England Human Research Ethics Committee considered the implementation of the PAST protocol and associated data collection as a quality assurance/improvement project and provided an exemption from full ethical review (10). Only routinely collected or published summary data have been used in this subsequent economic evaluation of the PAST protocol. Therefore, consent from patients in the control group was not needed based on a waiver of consent to use the de-identified administrative data for secondary analysis purposes.

### Primary and Secondary Outcomes

The primary outcome was the incremental cost of the intervention compared with the standard historical care (control). The secondary outcomes are health gain, measured by disability adjusted life years (DALYs), simulated for each patient with ischemic stroke in the control and intervention periods. The incremental cost per DALY averted in the intervention period was compared to the control period.

### Calculation of DALYs

The incremental DALYs averted from tPA treatment were calculated using estimates previously established from the model of resource utilization, costs, and outcomes for stroke (MORUCOS) model (12). The average incremental DALY averted per case over a lifetime of 0.4575 for stroke unit treatment over general management; and 0.605 for intravenous thrombolysis was applied to this cohort (13). Estimating the health benefits from both treatments was relevant because at JHH, treatment in a stroke unit was provided to all bypassed patients irrespective of whether they received tPA or not.

Patient discharge destinations (home, in-patient rehabilitation, nursing home, and in-hospital death) were extracted from the hospital administrative databases and the Hunter Region Heart and Stroke Outcomes Registry (14).

### Stroke Severity

The National Institutes of Health Stroke Scale (NIHSS) was used were available within the routinely collected clinical data. Scandinavian Stroke Scale was also available in the routine clinical data and converted to the NIHSS performed using the methods described by the Virtual International Stroke Trials Archive Collaboration (15).

The final eligibility of the study population was determined using medical records coding (International Classification of Diseases, 10th version). The specific ICD codes were: I60 (subarachnoid hemorrhage), I61 (intracerebral hemorrhage), I63 (ischemic stroke), I64 (undefined stroke), and G45 [code for transient ischemic attack (TIA)] (16, 17).

Inclusion criteria included all suspected stroke patients brought to JHH under the PAST protocol. Confirmation of acute ischemic stroke was diagnosed and tPA eligibility determined by the neurologists at the JHH. There were no exclusion criteria.

### Cost Data

Estimates were made of the resource utilization that occurs as part of the acute stroke care pathway using the PAST protocol.

**Figure 1:**
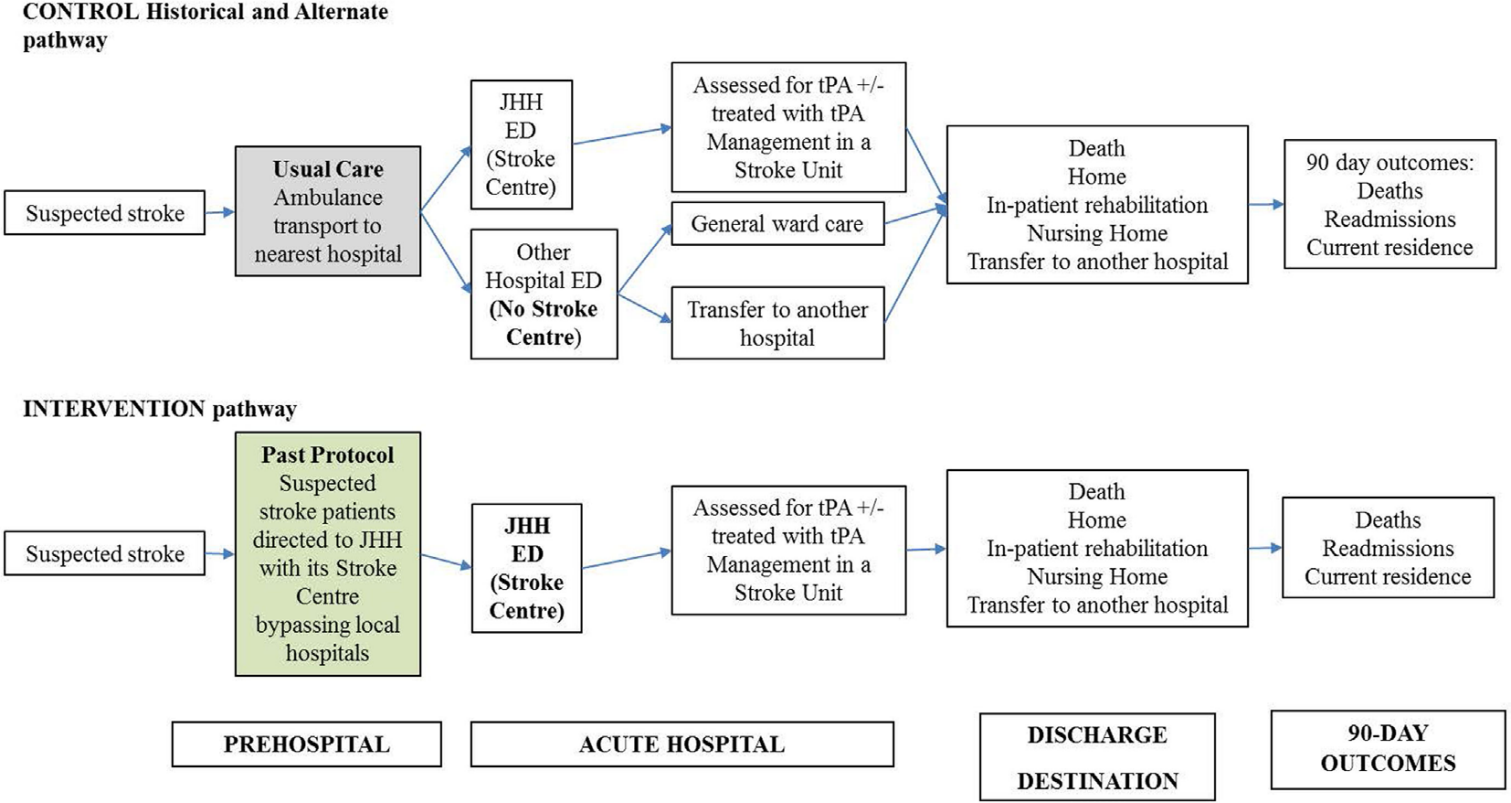
Decision analytic model for acute stroke care pathway using pre-hospital acute stroke triage (PAST) protocol compared to pre-PAST traditional management. ED: emergency department; CT: computed tomography; CTA: computed tomography angiography; JHH: John Hunter Hospital; NIHSS: National Institutes of Health Scale Score; t-PA: tissue plasminogen activator mediation.

Cost estimates were calculated using summary estimates of patient data from the both study cohort and local administrative data. For some of the unit prices, published national sources are used, where study or administrative data were unavailable. Total cost was calculated by multiplying the probability of resource used by the unit cost. The broad cost components are pre-hospital (transport costs), acute hospital (imaging and treatment), and post-hospital (rehabilitation and community-based resource use, including nursing home care and readmissions within 90 days) (Figure 1; Table A2 in Appendix).

### Currency and Reference Year for Costs

All costs were converted to reference year (2014) values using the total health price index (18). All costs were calculated in Australian dollars.

### Assumptions

The main modeling assumptions were:

1. Mode of transport to hospital was by road in ambulance in the control and intervention periods.
2. Apart from the PAST protocol, we assumed that the operating policies and procedures used by the acute stroke team were standard and did not alter over the period of the evaluation.

### Calculation of the Incremental Cost-Effectiveness Ratio

Cost per DALY averted was calculated using the following formula: Net average cost/DALY averted = (average cost per patient intervention group−average cost per patient combined control + alternate pathway group)/(average DALY averted per patient intervention group minus average DALY averted per patient combined control + alternated pathway group).

### Statistical Analyses

All calculations were undertaken using a computer-based model developed in Microsoft Excel. STATA was used to provide summary estimates from cohort data as inputs to the model, as relevant. Average cost is total cost divided by the total number of patients. Incremental cost is the difference in cost between the control and intervention periods.

Comparisons of patient outcomes between groups were conducted using Chi-Square, Kruskal–Wallis, or ANOVA tests, with *P*-values for comparisons set at 0.05 as calculated using STATA.

## Results

When the PAST protocol was implemented in a real-world setting enrollment of patients based on the inclusion criteria was initially slow, therefore, we collected 3 years of data for this cohort to more closely match the number in the control cohort so that the comparisons would be more reliable.

There were 364 patients with suspected stroke treated in the Hunter region’s hospitals during the historical control period and 309 patients in the same hospitals during the intervention period. Of the 364 historical control patients, 203 had an ischemic stroke (IS). Of the 309 intervention patients, 217 had an ischemic stroke (Table 1). Patients in the intervention group were more likely to have had an ischemic stroke compared to the historical control group (70 vs 56%, *p* < 0.01). There were no differences between the intervention and control groups in terms of age (median 77 years old) and gender (52% male). However, the intervention group had a mean NIHSS of 13.54 ± 7.43 compared to 8.05 ± 6.21 in the control group (*p* < 0.01). Considering the ischemic patients only, the intervention group had a mean NIHSS of 14.11 ± 6.64 compared to 8.28 ± 5.76 in the control group (*p* < 0.01). NIHSS data for the alternate control pathway were not available. The 5.88 difference between NIHSS scores of the intervention and historical control groups was statistically significant, indicating more severe stroke patients were transferred to JHH during the intervention period.

**Table 1:** Distribution of stroke subtypes.

Stroke subtype	Control*n*(%)	Alternate pathway*n*(%)	Intervention*n*(%)	*P* value^a^
Ischemic	203 (56)	123 (66)	217 (70)	<0.05
Lacunar infarct	41 (11)	14 (7)	18 (6)	<0.05
Partial anterior circulation infarct	65 (18)	39 (21)	75 (24)	<0.05
Posterior circulation infarct	33 (9)	6 (3)	11 (4)	<0.05
Total anterior circulation infarct	49 (13)	64 (34)	113 (37)	<0.05
Not specified	15 (4)	0 (0)	0 (0)	<0.05
Haemorrhagic	46 (13)	30 (16)	43 (14)	0.626
Undetermined	12 (3)	0 (0)	0 (0)	<0.05
Non-stroke	30 (8)	24 (13)	32 (10)	0.345
Transient ischemic attack	73 (20)	10 (5)	17 (6)	<0.05

Total	364	187	309	

*^a^Compares control and intervention*.

Of the 203 control ischemic stroke patients, 13 (6.40%) were thrombolysed, compared with 120 (55%) of the 217 intervention patients with ischemic stroke (Table 2). The equivalent participants in the alternate pathway were assumed not to have been provided thrombolysis based on the absence of thrombolysis availability in their destination hospitals.

**Table 2:** Proportion of patients with ischemic stroke receiving thrombolysis.

Control			Alternate pathway			Intervention			*P* value
Total ischemic stroke	Received tissue plasminogen activator (tPA)	%	Total ischemic stroke	Received tPA	%	Total ischemic stroke	Received tPA	%	
203	13	6	123	0	0	217	120	55	<0.05

The difference in the proportion of patients with ischemic stroke receiving thrombolysis between the historical control and intervention group was statistically significant (*p* < 0.05). Patients in the intervention group had a 17 times greater odds of receiving thrombolysis than patients in the control group (95% CI 9.42–31.2, *p* < 0.05).

A significantly greater proportion of patients in the control group were discharged home from hospital compared to that of the intervention group (48 vs 29% with IS patients and 49 vs 34% in all stroke patients, *p* < 0.05) (Tables 3 and 4). As expected, given the greater severity of stroke, a significantly greater proportion of patients in the intervention group died in hospital (22%) compared to the historical control group (8%, *p* < 0.05). The average time to death in the control group was greater than in the intervention group (*p* < 0.01) (Table 4). The age distribution was similar in both the intervention and control groups.

**Table 3:** Discharge destination of patients with ischemic stroke.

Discharge destination	Control *n*(%)	Alternate pathway *n*(%)	Intervention *n*(%)	*P* value^a^
Alive				
Home	98 (48)	37 (30)	64 (29)	<0.05
In-patient rehab	70 (34)	44 (36)	80 (37)	>0.05
Nursing home/other hostel	17 (8)	12 (10)	18 (8)	>0.05
Transfer to another hospital	3 (1)	10 (8)	12 (6)	<0.05
Unknown	3 (1)	0 (0)	0 (0)	>0.05
Death in hospital	12 (6)	20 (16)	43 (20)	<0.05

Total	203	123	217	

*^a^Compares control and intervention*.

**Table 4:** Discharge destination of all patients.

Discharge destination	Control *n*(%)	Alternate pathway *n*(%)	Intervention *n*(%)	*P* value^a^
Alive				
Home	180 (49)	64 (34)	105 (34)	<0.05
In-patient rehab	88 (24)	55 (29)	96 (31)	<0.05
Nursing home/other hostel	25 (7)	13 (7)	21 (7)	>0.05
Transfer to another hospital	9 (2)	17 (9)	20 (6)	<0.05
Unknown	32 (9)	0 (0)	0 (0)	<0.05
Death in hospital	30 (8)	38 (20)	67 (22)	<0.05
Mean time to death (in days)	11.9 ± 14.58	5.74 ± 6.18	6 ± 6.29	<0.01

Total	364	187	309	

*^a^Compares control and intervention*.

Of those alive at discharge, there was no difference (*p* > 0.05) between control and intervention group in terms of average length of stay (12 days for the control and 10 days for the intervention group).

Most costs were incurred from the acute hospital care and rehabilitation resource use. Total (pre-hospital, acute hospital, and post-hospital) costs were greater in the combined control group than the intervention group. Compared to control, the incremental average total cost per patient was $3,421 in the intervention group (Table 5). In the intervention group, it was estimated that 101.2 DALYs were averted related to the provision of tPA and stroke unit care. This compares to the estimated 7.9 DALYS in the combined control group. Overall, the intervention was associated with a net avoidance of 93.3 DALYs.

**Table 5:** Comparison of costs and health benefits.

Cost category	Intervention	Control	Alternate pathway	Combined control	Net difference^a^
	
	*a*	*b*	*c*	*b* + *c* = *d*	*d*−*a*

	*n* = 309	*n* = 364	*n* = 187	*n* = 551	
Pre hospital	$71.12	$53.71	$56.70	$54.72	−$16.40
Acute hospital	$10,275.15	$10,375.88	$9,466.36	$10,067.21	−$207.94
Imaging	$649.58	$179.87	$209.76	$190.01	−$459.57
Treatment	$1,882.63	$173.13	-	$114.38	−$1,768.25
Rehabilitation	$7,775.02	$6,950.35	$4,483.07	$6,113.00	−$1,662.02
Readmissions within 90 days	$1,141.68	$627.11	$2,564.58	$1,284.66	$142.98
Nursing home costs	$725.82	$699.45	$474.39	$623.07	−$102.76
Extension of hospital care^a^	$1,995.10	$705.52	$6,428.39	$2,647.76	$652.66
Average total cost/patient	$24,516.10	$19,765.03	$23,683.25	$21,094.81	$3,421.29
Total health benefits (DALYs avoided)	101.2	7.9	0	7.9	93.3
Total pathway cost	$7,575,474.36	$7,194,469.81	$4,428,767.99	$11,623,237.80	−$4,047,763.44

*DALY, disability adjusted life year*.

*Costs in AUD reference year 2014*.

*^a^Costs of additional hospital care when patients are transferred or return to hospital from rehabilitation*.

The intervention appears to be cost-effective, with the estimated cost per DALY averted per patient in the intervention group, compared to the combined control group, of $10,921 [$3421/(101.2 DALYs/309 patients)–(7.9 DALYs/551 patients)] across both the hospital and post-hospital phases of care.

## Discussion

Our study suggests that the implementation of the PAST protocol is a cost-effective intervention to improve access to evidence-based, effective stroke care. The implementation of the PAST protocol resulted in a significant increase in the rate of tPA treatment for patients with acute ischemic stroke. Overall, the incremental resource use at each stage of the clinical pathways projected within the economic model, suggesting potential opportunity cost savings of $4 million for the Hunter region over a 12-month period. Average pre-hospital (transport) costs were the lowest ($71.12) among all incurred cost items associated with the intervention. It is noteworthy that an economic evaluation of helicopter-transfer of patients with suspected acute ischemic stroke for potential thrombolysis showed that cost-effectiveness is not sensitive to the range of transport costs (19).

We are unaware of any studies that have previously addressed the cost-effectiveness of a similar intervention in the Australian healthcare setting. Our study provides an estimate of the cost per DALY averted and found 101.2 DALYs were potentially averted due to the implementation of the PAST protocol facilitating access to reperfusion therapy and stroke unit care. This compares to 7.9 in the combined control group; the intervention was, therefore, associated with a net avoidance of 93.3 DALYs compared to care provided in the control group. The intervention was cost-effective, with the net cost per DALY avoided per patient in the intervention group compared to the combined control group of $10,921.

There is a difference in point estimates regarding cost per DALY averted between our study (which showed additional cost per DALY averted due to the intervention) and MORUCOS (which showed less cost per DALY averted due to tPA) (Table 5) (12). This is because our study included an additional cost bucket, that is, the costs attributable to the implementation of the PAST protocol. Despite this difference, the additional cost was modest at $3,421 per patient (Table 5).

The implementation of the PAST protocol appears to be a feasible mechanism to ensure those who live at an extended distance from an acute stroke unit can also access effective stroke treatments (and their subsequent benefit) that are not otherwise available. This is an important issue of equity in healthcare for rural and regional Australia, as well as in other countries with regional and rural populations living at a distance from a Stroke Center with tPA availability. Other reliable, valid, efficacious, and safe mechanisms, such as Telestroke, are also found to be clinically effective and cost-effective in the pre-hospital setting (11).

The analysis of the data for discharge destination does not directly support benefit in the bypassed patients, but this may be due to case-mix difference in stroke severity between the intervention group and the comparator patients. The significantly greater proportion of in-hospital death of patients in the intervention group (22%) compared to in the control group (8%, *p* < 0.05), is a likely consequence of the greater severity of stroke as described by the average difference in NIHSS scores of 5.83 points. Economic evaluation of stroke care studies show severity of stroke has a major influence on stroke outcome (20, 21). A limitation of this study is that we are unable to completely adjust for patient case-mix given the indirect assumptions made for the alternate care pathway. It is understandable, therefore, that despite higher tPA rates, our study showed higher in-hospital case fatality rate and shorter time to death in the intervention group along with a lower proportion of patients in the intervention group discharged home.

A consequence of this limitation is that the reported economic results reported in this study may be biased toward being unduly conservative with the results underestimating the intervention’s improvement in DALYs averted and overestimating the cost per DALY averted. This is because there were more severe patients in the intervention group. Therefore, DALYs averted due to the intervention in this study are likely to be lower than would have occurred if stroke severity was equivalent in the control and intervention groups.

Regarding hospital stay, there was no statistically significant difference between the control and intervention groups.

The economic findings are in accordance with international pre-hospital economic evaluation studies (3, 19–21), showing that acute stroke care, starting in the pre-hospital phase, can reduce cost, reduce the time to initiate tPA for eligible stroke cases, and thus increase the likelihood of eligible patients receiving access to effective stroke treatment.

The strength of our study is the provision of an estimate of the potential cost per DALY averted as a consequence of the PAST protocol. There are very few published economic evaluations of transport protocols in stroke care nationally or internationally. This is the first study on the pre-hospital stroke protocol to our knowledge and, therefore, internationally relevant. An economic evaluation of helicopter-transfer of patients with suspected acute ischemic stroke for potential thrombolysis showed that cost-effectiveness is not sensitive to the range of transport costs and other system variables (19).

A limitation of this study is that we used indirect data and summary measures from a previous study for the calculation of DALYs and, hence, not derived directly from patient deaths and disability. Likewise, lack of availability of empirical data lead us to develop a cost model using probability of resource use for an event. Therefore, the cost and DALYs calculated here are simulated estimations.

## Conclusion

In this simulation study, the implementation of the PAST protocol in regional Australia was found to be a potentially cost-effective strategy to improve tPA rates among eligible ischemic stroke patients. The likely cause of higher death and shorter time to death in the intervention group is the greater severity of stroke. The evidence indicates that for a modest increase in cost ($3,421) per patient there is a significant aversion of DALYs. To our knowledge, this is the first Australian study to reveal the potential resource implications and patient outcomes of protocols designed to increase equitable access to tPA followed by care in stroke units among eligible ischemic patients.

## Author Contributions

For the manuscript entitled: “Economic evaluation of a protocol to reduce time between event and treatment for patients with suspected acute stroke,” SL has substantially contributed to: conceiving the research question and study design; conducting literature search; performing of primary statistical analysis; and preparing the manuscript. CL: formulating the aims and hypothesis; contributing to the epidemiological and economic methodology; and academic and policy related discussion. JK: performing of statistical analysis and disability adjusted life years (DALY) calculation. DC: contribution to study design, development of the economic model, souring some of the administrative data, drafting of the manuscript and interpretation. AS: detailing of the methodology, quality improvement, and policy-related recommendations.

## Conflict of Interest Statement

The authors declare that the research was conducted in the absence of any commercial or financial relationships that could be construed as a potential conflict of interest.

## Appendix

**Table A1:** Estimates of intravenous thrombolysis in acute stroke patients.

Country	Tissue plasminogen activator rate	Reference
Australia	7% of all ischemic stroke patients in 2013	NSF Audit Report (22)

Canada	6.1% of all ischemic stroke (i.e., 5.4% of all stroke) patients from audit sample (2008 and 2009) in 2013	Ganesh et al. (23)

England, Wales, and Northern Ireland	12.2% of all acute ischemic stroke patients in 2014 (this was 1.8% in 2008)	Jing et al. (24), State of the Nation, Stroke statistics, Stroke Statistics (16)

India and China	Although two-thirds of the strokes happen in India and China (WHO Atlas 2004), there is no comprehensive statistics on tPA rate	Mehndiratta et al. (25)

Japan	5% of acute ischemic stroke cases in 2012	Kunisawa et al. (26)

Scotland	9% of all acute ischemic stroke patients in 2014	Stroke statistics, stroke.org.uk (16)

United Kingdom	Around 15% of stroke emergencies in UK are eligible for thrombolysis treatment on admission to hospital	Stroke statistics, stroke.org.uk (16); American Heart Association (7)

United States of America	10% of all patients in 2014	American Heart Association (7, 17)

**Table A2:** Cost category, matrix, source of data, and comments on the data of the study population.

Cost category	Matrix	Source of data	Comments
Pre hospital	Ambulance data including paramedics	Manager, Aeromedical, and Retrieval Services, Ambulance Service New South Wales, National Health Expenditure (NHE) Administration	This dataset did not contain data on number of patients transported *via* ambulance in the control period. In the pre-period, we assumed 76% came to hospital *via* ambulance, where these data were not available as per the NSF audit report. We assumed all patients who received the intervention were taken by ambulance

Acute hospital	Imaging, treatment, nursing, in-patient costs for length of stay	NHE Administration, thrombolysis, and nursing cost from Gosford hospital data	Costs for tissue plasminogen activator were not available from John Hunter Hospital (JHH) and so these were extrapolated from Gosford Hospital (GH). But at the time of data collection, GH provided with more complete data than JHH^a^

Post-hospital Rehabilitation	Home, nursing home, other hospitals	NHE Administration	JHH data

Readmissions within 90 days	Imaging, treatment, in-patient costs for length of stay	NHE Administration, the basic protocol for hospital patient care	JHH data

*^a^Most transfers may have occurred and not taken into consideration*.
